# MXene-Derived Multifunctional Biomaterials: New Opportunities for Wound Healing

**DOI:** 10.34133/bmr.0143

**Published:** 2025-02-11

**Authors:** Dong Luo, Hui-Qi Zhang, Xin-Yang Xuanyuan, Dan Deng, Zheng-Mao Lu, Wen-Shang Liu, Meng Li

**Affiliations:** ^1^ Department of Dermatology, Shanghai Children’s Medical Center, School of Medicine, Shanghai Jiao Tong University, Shanghai 200127, People’s Republic of China.; ^2^ Department of Dermatology, Shanghai Changhai Hospital, Naval Medical University, Shanghai 200433, People’s Republic of China.; ^3^ Department of Gastrointestinal Surgery, Shanghai Changhai Hospital, Naval Medical University, Shanghai 200433, People’s Republic of China.

## Abstract

The process of wound healing is frequently impeded by metabolic imbalances within the wound microenvironment. MXenes exhibit exceptional biocompatibility, biodegradability, photothermal conversion efficiency, conductivity, and adaptable surface functionalization, demonstrating marked potential in the development of multifunctional platforms for wound healing. Moreover, the integration of MXenes with other bioactive nanomaterials has been shown to enhance their therapeutic efficacy, paving the way for innovative approaches to wound healing. In this review, we provide a systematic exposition of the mechanisms through which MXenes facilitate wound healing and offer a comprehensive analysis of the current research landscape on MXene-based multifunctional bioactive composites in this field. By delving into the latest scientific discoveries, we identify the existing challenges and potential future trajectories for the advancement of MXenes. Our comprehensive evaluation aims to provide insightful guidance for the formulation of more effective wound healing strategies.

## Introduction

Injuries have garnered considerable attention in the global health sector [1,2]. When the skin is injured, harmful factors such as severe bacterial infections [3], oxidative stress [4], and ischemia [5] can disrupt the metabolic balance within the wound environment, thereby hindering the healing process [6]. Addressing these metabolic imbalances at the wound site is a considerable challenge. Recently, 2-dimensional (2D) multifunctional bioactive nanomaterials have demonstrated great promise in wound healing due to their adaptable structures and diverse properties [7,8]. MXenes, a new class of 2D nanomaterials, have retained the key advantages of traditional nanomaterials, such as ultrathin thickness, a large specific surface area, and excellent mechanical strength. In addition, they possess remarkable physical and chemical properties, including exceptional electrical conductivity, good hydrophilicity, and unique photothermal conversion capabilities. These characteristics make MXenes an outstanding candidate for wound healing applications [9,10].

MXenes were first introduced by researchers Gogotsi and Barsoum in 2011 [11]. They are characterized by a chemical formula of M_*n*+1_X*_n_*T*_x_*, where “M” represents early transition metal elements (Ti, V, Nb, Mo, etc.), “X” denotes C or N, and “T*_x_*” refers to the functional groups on the surface of MXenes (-OH, -O, or -F, etc.) [12]. These materials are incredibly thin, with a thickness of a single atom or a few atoms (typically less than 5 nm), while their lateral dimensions can span over 100 nm, reaching several micrometers or more [13]. The remarkable photothermal and photodynamic effects of MXenes have been instrumental in combating bacterial infections and hastening the wound healing process. Their superior electrical conductivity has also opened up new avenues for advancements in vascular regeneration and tissue repair [14,15]. The ease with which MXenes can be surface functionalized enables the customization of bioactive materials to meet specific therapeutic requirements [16,17]. However, MXenes are not without their challenges; they may struggle to conform to the complex geometry of wounds and are prone to oxidation and degradation in air, and their therapeutic effects may be short-lived [7]. Fortunately, the combination of MXenes with other nanomaterials, including hydrogels, microneedles (MNs), hemostatic sponges, and nanofibrous membranes, has shown promise as an effective strategy [18,19]. This synergistic approach not only capitalizes on the unique attributes of MXenes but also enhances the overall wound healing effectiveness by overcoming the limitations of individual materials [20].

**Fig. 1.**
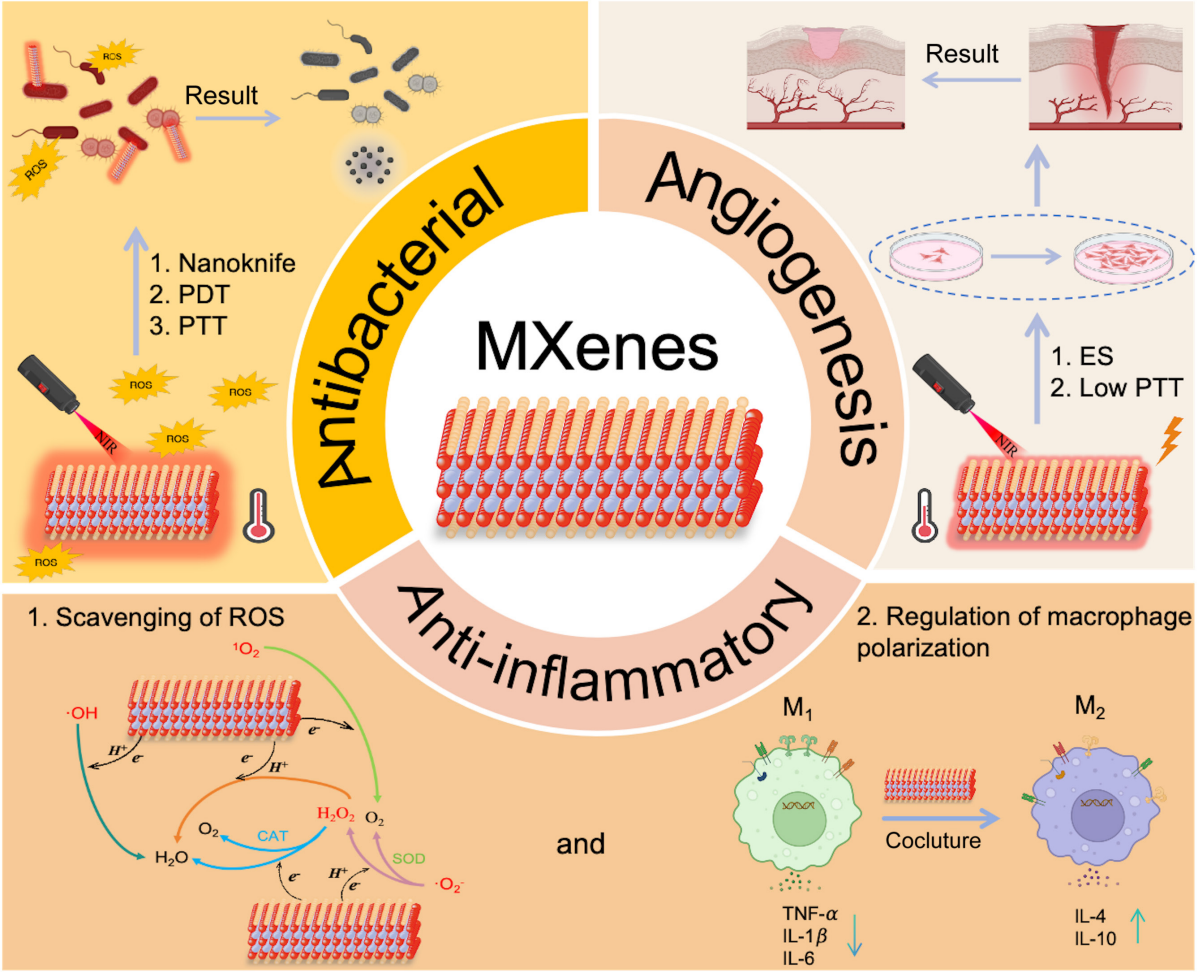
Schematic illustration of the mechanisms by which MXenes promote wound healing created via BioRender.com.

According to our survey, the existing research on MXenes is primarily concentrated on its synthesis, characterization, and intrinsic properties, with relatively scant engagement with its biomedical applications [21–23]. In particular, there is a conspicuous deficiency in exhaustive analysis and comprehensive summarization concerning the role of MXenes in wound healing. Therefore, this review aims to bridge this knowledge gap by outlining design strategies for MXenes and offering an in-depth exploration of their mechanisms that expedite the wound healing process. Figure 1 visually presents the various mechanisms of action of MXenes, including antibacterial, anti-inflammatory, and angiogenic effects. Moreover, this article also encapsulates the most recent findings on MXene-based multifunctional bioactive composites for wound care, spotlighting the challenges encountered in real-world applications and suggesting possible remedies. Looking ahead, the continuous refinement of our understanding and utilization of MXene properties is poised to herald a new era of more potent and inventive therapeutic approaches, thereby pushing the boundaries of wound management.

## Design Strategies for MXenes

### Synthesis Strategies for MXenes

MAX phases, the progenitors of MXenes, are known for their ternary layered structure, which shares a similar stoichiometry with MXenes, expressed as M_*n*+1_AX*_n_*, where “A” stands for elements such as Al, Si, and Sn [24,25]. The M–A bonds in the MAX phase are metallic and exhibit a higher chemical reactivity compared to M–X bonds, which allows for the selective chemical etching of “A” atoms [26]. The synthesis of MXenes is primarily conducted through a top-down strategy, typically divided into 2 distinct stages: The first stage involves the selective etching of “A” atomic layers from the MAX phase precursor, resulting in multilayered MXenes with a distinctive accordion-like structure [27]; the second stage focuses on the exfoliation of these multilayers to obtain single-layered MXenes (Fig. S1A) [28,29]. A plethora of methods for the synthesis of MXenes have been documented, each with its inherent strengths and limitations (Table S1) [30–37]. Consequently, the choice of an etching method for the production of high-quality MXenes should be guided by a comprehensive set of factors, including material properties, preparation protocols, and operational conditions.

### Properties of MXenes

The exquisite structural design of MXenes confers upon them a suite of highly adjustable properties. These extraordinary characteristics have shown unprecedented application prospects and tremendous potential in the critical biomedical field of wound healing [23,38]. This section will focus on an in-depth analysis of the hydrophilicity, biocompatibility, biodegradability, and conductivity of MXenes [39,40]. The optical properties of MXenes will be thoroughly addressed in subsequent sections.

#### Hydrophilicity

In the realm of biomedicine, the hydrophilicity of materials plays a pivotal role in modulating cell behavior, including adhesion, proliferation, and differentiation [39]. The presence of functional groups on the surface of MXenes enables them to form hydrogen bonds with water molecules, thereby endowing MXenes with unique hydrophilicity. Recent studies have shown that the hydrophilic properties of MXenes are closely related to the type and distribution of their surface functional groups [41]. As MXenes transition from fluorine–oxygen termini to hydroxyl termini, their water absorption capacity increases significantly (Fig. S1B). The cause of this phenomenon may be that the fluorine and oxygen termini can act as weak hydrogen bond acceptors, while the hydroxyl groups can also function as donors, enhancing the hydrogen bonding interaction with water molecules. Additionally, vacancies at metal sites have become a focus of research. Researchers have found that individual surface vacancies can importantly enhance the adsorption reactivity of MXenes to water molecules [41]. However, for carbon/nitrogen vacancies and titanium vacancies present in bulk MXenes, their adsorption behavior toward water molecules does not seem to markedly deviate from that of defect-free MXenes, highlighting the variability of vacancy effects at different scales. In summary, the hydrophilicity of MXenes not only depends on their inherent surface functional groups but also is profoundly influenced by microscopic structural features such as surface vacancies. By finely tuning these factors, we can customize MXene materials with specific hydrophilic properties to meet the demands for high-performance biomaterials in the biomedicine field.

#### Biocompatibility

The good biocompatibility of MXenes is a prerequisite for their application in the field of wound healing [42]. This characteristic is subject to precise regulation by multiple factors, including nanoscale size, dosage, and surface modification. Through meticulous in vitro experimental designs, the Scheibe team has profoundly revealed the dual impact of the size and dosage of Ti_3_C_2_T*_x_* MXenes on their biological effects (Fig. S1C) [43]. Specifically, at concentrations ranging from 10 to 400 μg/ml, MXenes exhibit a clear dose-dependent cytotoxicity trend, while their compatibility with nonmalignant cells remains at a high level of over 80%. Notably, when the size of MXenes is refined to less than 44 μm, their subtle influence on the vitality of normal cells is further revealed, highlighting the pivotal role of size control in optimizing the biological performance of MXenes. Furthermore, the research by Hussein et al. [44] has demonstrated a new avenue to enhance the biosafety of MXenes through composite strategies. They ingeniously combined Au, Fe_3_O_4_ with Ti_3_C_2_ MXene, creating an Au/Fe_3_O_4_/MXene composite that exhibits significantly lower toxicity in both in vitro and in vivo experiments [with a median lethal concentration (LC_50_) value notably increased to over 1,000 μg/ml, compared to 257.46 μg/ml for pure MXene]. This achievement not only broadens the application horizons of MXenes but also indicates that by rationally designing composite materials, the biological effects of nanomaterials can be effectively regulated, thereby achieving higher biosafety and broader application potential.

#### Biodegradability

The excellent biodegradability of biomaterials signifies that they can gradually be absorbed or decomposed by the biological organism during application, without leaving persistent residues in the body, thereby averting potential foreign body reactions or rejection responses [45]. Chen’s research group synthesized an ultrathin Mo_2_C MXene treated with polyvinyl alcohol (PVA) and conducted an in-depth study on its biodegradability [46]. Experimental results indicated that Mo_2_C-PVA exhibited significant pH-dependent degradation behavior. The Mo_2_C-PVA nanosheets degraded rapidly under alkaline conditions and were relatively stable under acidic conditions. Furthermore, detailed transmission electron microscopy (TEM) observations revealed that after incubation in phosphate-buffered saline (PBS) solution at pH 7.4 for 48 h, Mo_2_C-PVA nanosheets completely decomposed into some ultrasmall particles (Fig. S1D). Notably, the presence of hydrogen peroxide can accelerate the degradation of Mo_2_C-PVA nanosheets (Fig. S1E). The reason may be that its strong oxidation property can accelerate the chemical bond breaking in MXene material, thus further promoting the decomposition process of the material.

#### Electrical conductivity

Electrical stimulation (ES) therapy, which harnesses the power of minuscule currents or electric fields to promote cell proliferation, migration, and differentiation, has sparked considerable interest in the domain of wound healing. The layered structure of MXenes, composed of metal atoms with carbon or nitrogen atoms, confers these materials with marked electrical conductivity, positioning them as a promising medium for ES therapy [47]. Through meticulously designed surface modification, ion intercalation, and morphological control strategies, the band structure and electrical conductivity of MXenes can be importantly optimized and adjusted. Intercalating specific ions (such as Li^+^, Na^+^, and K^+^) between the layers of MXenes not only effectively increases the carrier density, thereby enhancing the electrical conductivity of MXenes, but also provides a flexible means for performance regulation. However, it is important to note that when large-volume cations are introduced between the MXene layers, although the expansion of the interlayer distance may bring about some unique physical effects, it may also suppress the electronic hopping process between the layers, thereby inducing a transition from metallic to semiconductor behavior (Fig. S1F) [48]. In addition, the electrical conductivity of MXenes is closely related to their microstructure. For example, ultrathin, dense MXene films, when their structure exhibits a highly ordered arrangement of flakes, can demonstrate exceptionally good electrical conductivity, with a conductivity value as high as 15,100 S/cm. In contrast, when MXenes exist in the form of aerogels, their highly porous structural characteristics make the electron transport path complex and inefficient, leading to a significant reduction in conductivity, dropping by 2 orders of magnitude compared to dense films. This phenomenon deeply reveals the intrinsic connection between the electrical conductivity and the morphological structure of MXenes, providing valuable insights for the targeted design and application of MXene materials.

## Potential Mechanisms by which MXenes Promote Wound Healing

Wound healing is a complex and intricately coordinated process that involves a series of stages: hemostasis, inflammation, proliferation, and remodeling. Each of these stages is essential and interconnected, forming a continuous cycle of healing [49]. Perturbations at any stage can disrupt this process, leading to protracted healing times or, in adverse cases, treatment failure [50]. MXenes, recognized for their multifunctional bioactivity, leverage a suite of unique properties to bolster the wound healing process through diverse biological pathways [7,20]. This section offers a comprehensive examination of the mechanisms underlying MXenes’ antibacterial, anti-inflammatory, and pro-angiogenic properties, clarifying their pivotal role in facilitating the wound healing process.

### Antibacterial activities

Bacterial infections pose an important barrier within the wound healing process and are capable of escalating local inflammation to detrimental levels [51]. If unchecked, this exacerbated inflammatory response can lead to bacteremia, potentially progressing to sepsis and systemic infection [52–54]. Thus, prompt and effective bacterial infection management is crucial in wound care and must be a central focus of clinical protocols to optimize healing outcomes. Traditional antibacterial agents are often limited by their singular mode of action, which may foster bacterial resistance over time [55]. Compared to conventional drugs, MXenes possess a multifaceted antibacterial mechanism, which is believed to be the reason for their stronger activity and lower propensity to induce bacterial resistance [56]. The reported antimicrobial mechanisms of MXenes are categorized into 3 primary modalities [57]: (a) direct physical interaction, (b) photodynamic therapy (PDT), and (c) photothermal therapy (PTT).

#### Physical contact

**Fig. 2.**
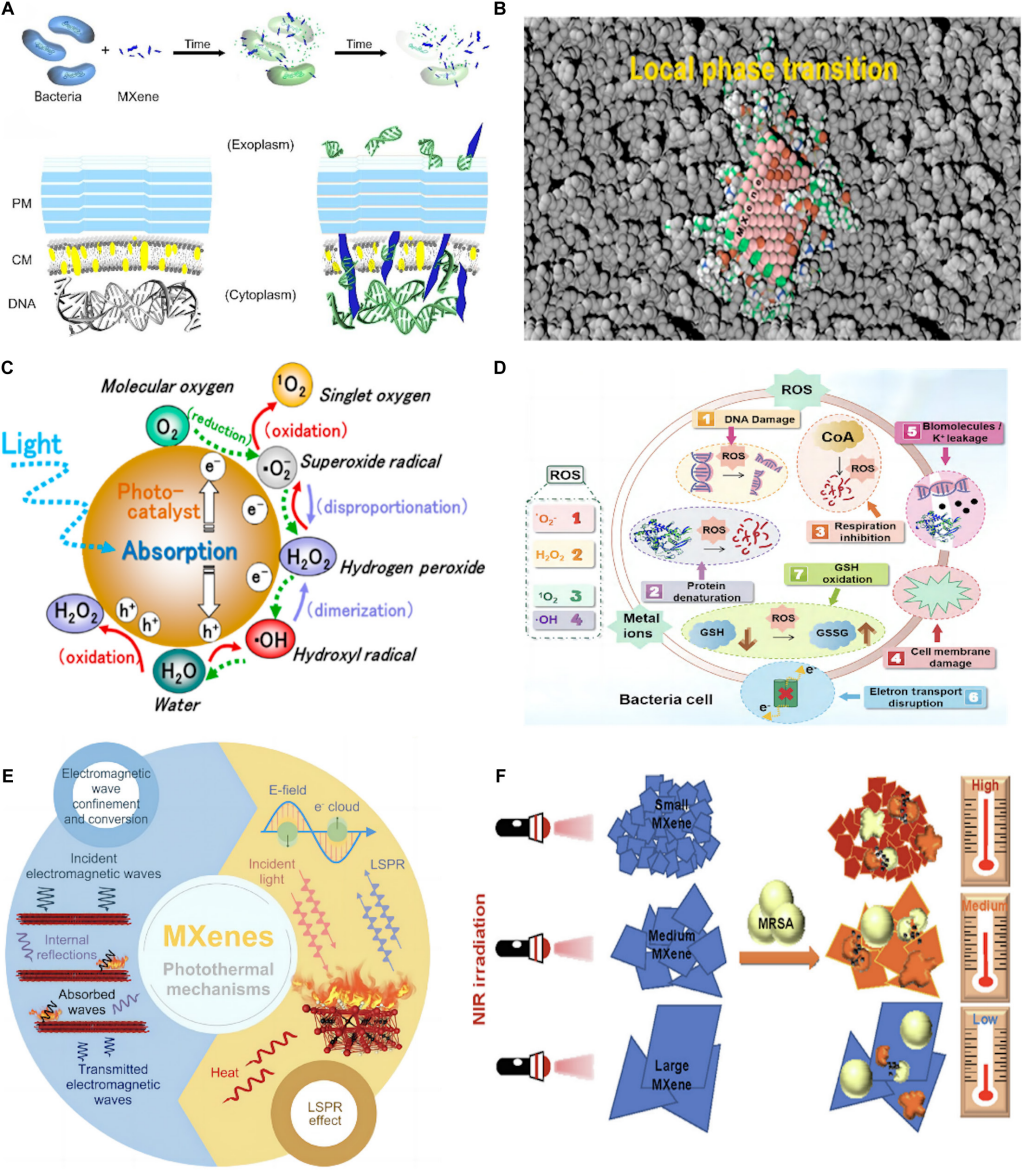
The antibacterial mechanisms of MXenes. (A) Schematic representation of the process by which the sharp MXene nanosheets lead to bacterial death by cutting the cell wall and cell membrane. PM and CM denote the peptidoglycan mesh and the cytoplasmic membrane, respectively. Reprinted from [58] with permission. Copyright 2018 American Chemical Society. (B) Schematic illustration depicting the molecular mechanism by which MXenes induce a localized phase transition on the surface of the cellular membrane. Reprinted from [60] with permission. Copyright 2021 American Chemical Society. (C) Schematic diagram of the generation of ROS by MXenes in the photocatalytic process through the redox reactions of O_2_ and H_2_O. Reprinted from [64] with permission. Copyright 2017 American Chemical Society. (D) Mechanisms of the effect of ROS on bacterial cells. Reprinted from [70] with permission. Copyright 2023 American Chemical Society. (E) Schematic illustration of the PTT mechanisms of MXenes. Reprinted from [72] with permission. Copyright 2022 Springer Nature. (F) Illustration of the size-dependent photothermal performance and antibacterial impact of Ti_3_C_2_ MXene on MRSA under NIR exposure. Reprinted from [82] with permission. Copyright 2022 Elsevier Inc.

MXenes, characterized by their exceptional hydrophilicity and sharp edges, perform a cutting action analogous to a blade. Upon engagement with bacterial cells, these edges can swiftly and effectively breach the bacterial cell wall and membrane. This physical intrusion triggers the release of cellular contents, leading to bacterial death (Fig. 2A) [58]. In 2016, Rasool et al. [59] pioneered the “Nanoknife” concept, utilizing scanning electron microscopy (SEM) and TEM to document the morphological alterations in *Escherichia coli* and *Bacillus subtilis* (*B. subtilis*) subsequent to their exposure to Ti_3_C_2_ MXene. The study demonstrated a pronounced interaction between Ti_3_C_2_ MXene and the bacterial surfaces, with a concentration of 100 μg ml^−1^ causing considerable damage to the cell membranes and subsequent cytoplasmic leakage. These findings robustly support the “Nanoknife” effect attributed to MXenes. To further decipher the antibacterial mechanisms of MXenes through physical contact, Lee et al. [60] provided molecular insights into this phenomenon. Molecular dynamics simulations illustrated that contact with bacterial cell membranes by Ti_3_C_2_ MXene incites rapid local phase changes (Fig. 2B). In the impacted area, phospholipids exhibited reduced fluidity and formed a thinner lipid bilayer compared to the surrounding phospholipids. Consequently, the diminished integrity of the bacterial cell membrane due to Ti_3_C_2_ MXene, precipitating the escape of intracellular material, is a critical element in the process of bacterial lysis.

A wealth of scientific literature has consistently shown that MXenes demonstrate superior antibacterial efficacy against gram-positive bacteria (G^+^) over gram-negative bacteria (G^−^) at lower concentrations [56,59]. This pronounced difference in susceptibility is largely due to the structural and compositional variations in the bacterial cell walls [61]. Specifically, the cell walls of G^−^ are encased by an outer membrane that includes a lipid bilayer, lipoproteins, and lipopolysaccharides (LPSs), which act as a shield, mitigating the impact of MXenes on the underlying cell membrane [62]. Moreover, G^−^ has a higher isoelectric point than G^+^ under the same conditions, endowing them with a more negatively charged cell surface [63]. This increased negative charge can engender a more potent electrostatic repulsion with negatively charged MXenes, potentially reducing the penetrative capacity of the “Nanoknife” effect.

#### PDT

Upon laser irradiation, MXenes activate charge carriers, leading to the formation of electron–hole pairs (e^−^/h^+^). The distinctive electronic band structure of MXenes, coupled with the effects of surface functional groups, facilitates the efficient separation of these pairs, paving the way for the generation of reactive oxygen species (ROS). In this process, water molecules (H_2_O) are oxidized by holes (h^+^) to produce hydroxyl radicals (•OH) and hydrogen peroxide (H_2_O_2_). Concurrently, molecular oxygen (O_2_) is reduced by electrons (e^−^) to form superoxide anions (•O_2_^−^), which then react with holes to yield singlet oxygen (^1^O_2_) (Fig. 2C) [64]. The generated ROS endowed with potent oxidative properties, can directly affect bacterial cell walls and membranes. This assault compromises the structural integrity of bacterial cells, triggering the release of intracellular contents and culminating in cell death [65]. In addition to the direct effects, ROS may also indirectly hasten bacterial apoptosis or necrosis through a series of deleterious actions. These include the induction of mitochondrial membrane potential depolarization, the induction of DNA damage, the promotion of protein denaturation, and the perturbation of the intracellular redox equilibrium (Fig. 2D) [66–70]. Wang et al. [71] cocultured the synthesized Ti_3_C_2_ MXene with *E. coli* and subsequently exposed the culture to brief laser irradiation. This procedure precipitated a notable increase in the levels of intracellular ROS. This substantial alteration unequivocally substantiates the capacity of Ti_3_C_2_ MXene to effectively neutralize bacterial populations through the generation of ROS.

#### PTT

The intricate layered structure of MXenes, in conjunction with their proficiency in electromagnetic wave absorption and conversion, and the notable localized surface plasmon resonance (LSPR) effect, endows these materials with exceptional photothermal conversion capabilities [72]. At the surface-active sites of MXenes, the formation of dipoles is initiated, adeptly capturing electromagnetic waves. In a synergistic manner, the reflective movements of free electrons and ions bolster the transformation of these waves into thermal energy [73]. Furthermore, the LSPR effect, induced by the resonance between incident photons and the free electrons within MXenes, markedly amplifies the efficiency of photothermal conversion (Fig. 2E) [74–76]. Li and his team [77] have successfully fabricated Ti_3_C_2_ MXene, which demonstrated a considerable temperature elevation to 70 °C within just 10 min of near-infrared (NIR) light exposure. This achievement underscores the superior photothermal conversion efficiency of Ti_3_C_2_ MXene, offering compelling evidence for their potential in antibacterial applications.

Within the intricate regulatory networks of bacteria, proteins such as DNA repair enzymes, proteases, and superoxide dismutases (SODs) are pivotal and acutely sensitive to temperature increases [78]. The elevated temperatures induced by MXenes can seriously disrupt the metabolic balance of bacteria, leading to the denaturation and damage of nucleic acids and proteins [78–80]. Consequently, the ability of bacteria subjected to MXene treatment to evolve drug resistance through metabolic adaptation or reduced uptake strategies is impeded [81]. Gao et al. [82] have showcased that Ti_3_C_2_ MXene, capitalizing on its exceptional photothermal efficiency, displays remarkable efficacy in curbing antibiotic-resistant bacteria. Notably, their research marks the first to delineate a direct link between the size of Ti_3_C_2_ MXene and their photothermal antibacterial potency. Employing ultrasonication technology, they meticulously crafted Ti_3_C_2_ MXene suspensions across a spectrum of sizes: small (MX-s, 196 nm), medium (MX-m, 347 nm), and large (MX-l, 497 nm). After 5 min of NIR irradiation, the diminutive MX-s suspension was observed to achieve the most pronounced temperature surge, correlating with the most robust suppression of methicillin-resistant *Staphylococcus aureus* (MRSA) biofilms. The pronounced size-dependent effect is hypothesized to stem from the heightened edge density of the smaller Ti_3_C_2_ MXene nanosheets, potentially augmenting their NIR light absorption and, by extension, their photothermal response (Fig. 2F). This investigation not only deepens our comprehension of the photothermal attributes of MXenes but also, more importantly, unveils the critical influence of size on their bactericidal efficacy from an innovative angle. The optimization of the size and shape of MXenes heralds a promising path toward the development of more potent and targeted photothermal antibacterial agents.

Although the antibacterial mechanisms of MXenes—physical contact, PTT, and PDT—are distinct, they are interwoven in a complex synergy that considerably amplifies the antimicrobial efficacy of MXenes [83]. The initial physical interaction with bacterial cells by MXenes sets the stage for damage, thereby creating an optimal environment for subsequent PTT and PDT actions. The localized heat generated by PTT boosts the kinetics of redox reactions, hastening the production of ROS. Owing to their high reactivity, these ROS assail bacterial cell walls and membranes, inflicting structural harm and inducing functional impairments. Moreover, bacteria with compromised structures are more susceptible to PTT, where the elevated temperatures further aggravate the cellular damage [72]. This synergistic intensification of antibacterial mechanisms underscores the profound benefits of MXenes in combating bacteria and also paves the way for innovative approaches and strategies in the creation of novel, potent antibacterial materials.

#### Enhanced PTT and PDT effects by MXene heterojunctions

MXenes form heterojunctions with distinct bandgaps and energy levels through covalent or noncovalent interactions with additional photosensitizers [84]. During the wound healing process, MXene heterostructures exhibit enhanced PTT and PDT effects, effectively killing bacteria and preventing wound infection. The Table offers an exhaustive overview of the various MXene heterojunctions that have been applied in wound healing [85–92].

**Table.** Types of MXene-based heterojunctions currently used for wound healing

Heterojunction types	Antimicrobial mechanism	Light source and treatment time	Types of bacteria	Effect	Ref.
Ti_3_C_2_/ZnTCPP	PDT/Zn^2+^	Visible light, 200 W, 10 min	*E. coli*	99.9%	[85]
			*S. aureus*	99.9%	
V_2_C/PtNPs	PTT/CDT/PDT	1,064 nm, 1.0 W cm^−2^, 5 min	*MRSA* biofilm	99.9%	[86]
V_2_C/BP	SPT	Ultrasonic irradiation, 1.5 W cm^−2^, 10 min	*E. coli*	98.2%	[87]
			*S. aureus*	98.8%	
			*DRB*	98.9%	
Ti_3_C_2_/CuS	PTT/PDT/Cu^2+^	980 nm, 1.4 W cm^−2^, 30 min	*E. coli*	99.9%	[88]
			*S. aureus*	99.9%	
Nb_2_C/Ag/PDA	PDT/PTT/Ag^+^	808 nm, 2.15 W cm^−2^, 10 min	*E. coli*	100%	[89]
			*S. aureus*	100%	
Ti_3_C_2_/Fe_2_O_3_	PTT/CDT/PDT/Fe^2+^	808 nm, 1.5 W cm^−2^, 10 min	*E. coli*	99.0%	[90]
			*S. aureus*	97.8%	
Ti_3_C_2_/Cu_2_O	PTT/PDT	808 nm, 0.54 W cm^−2^, 10 min	*E. coli*	>90%	[91]
			*S. aureus*		
			*MRSA*		
			*GREC*		
Ti_3_C_2_/Ag_3_PO_4_	PTT/PDT/Ag^+^	808 nm, 1.5 W cm^−2^, 10 min	*E. coli*	98.6%	[92]
			*S. aureus*	99.3%	

The exceptional antimicrobial potency of MXene heterojunctions is largely due to their unique structural features, which promote the efficient dissociation of e^−^ and h^+^, thus enhancing the photocatalytic production of ROS. Moreover, the integration of various photosensitizers with unique optical properties synergistically enhances the photothermal conversion efficiency of the heterojunction [93,94]. Li et al. [94] have successfully engineered an innovative Bi_2_S_3_/Ti_3_C_2_T*_x_* MXene heterojunction by integrating Bi_2_S_3_ with Ti_3_C_2_ MXene. This composite adeptly overcomes the limitations inherent in the solitary use of Ti_3_C_2_ MXene, particularly the inadequate generation of ROS and thermal energy (Fig. S2A and B). The Bi_2_S_3_/Ti_3_C_2_T*_x_* interface demonstrates the lowest charge transfer resistance coupled with the most rapid charge transfer kinetics. With prolonged irradiation, the Bi_2_S_3_/Ti_3_C_2_T*_x_* system shows a progressively enhanced generation of ROS, markedly surpassing the ROS production of pristine Ti_3_C_2_T*_x_*. Moreover, the photothermal conversion efficiency of the optimized Bi_2_S_3_/Ti_3_C_2_T*_x_* composition achieves an impressive 35.43%. Feng and his team [95] employed in situ sulfidation to craft a distinctive hollow, flower-like BiOI@Bi_2_S_3_/Ti_3_C_2_ ternary composite heterojunction on a 2D Ti_3_C_2_ MXene platform (Fig. S2C and D). This design enhances the material’s photothermal conversion efficiency to 57.8% under 808-nm laser irradiation and delivers exceptional antibacterial performance. This innovative strategy provides a new direction for heterojunction materials in the field of photothermal and photodynamic sterilization.

While heterojunctions combining metal or metal compound nanoparticles with MXenes may exhibit improved antimicrobial potency, their heavy metal content could potentially lead to toxicity and stability issues in clinical settings [96]. Conversely, organic photosensitizers are typically characterized by few immunological and toxicological reactions upon interaction with biological systems [97]. Therefore, merging organic photosensitizers with MXenes represents an auspicious strategy for enhancing the therapeutic effectiveness in wound healing and facilitating their clinical adoption. The judicious integration of porphyrins into MXenes to create ZnTCPP/Ti_3_C_2_T*_x_* heterojunction allows for the efficient initiation of photocatalytic activities under visible light irradiation, offering a straightforward and economical method to progress wound healing treatments (Fig. S2E) [85]. The ZnTCPP/Ti_3_C_2_Tx heterojunction displayed remarkable biocompatibility and potent antibacterial biofilm capabilities, contributing to the promotion of wound healing. The structural adaptability of organic photosensitizers provides a wide array of design possibilities for MXene heterojunctions. In the forthcoming years, the precise tuning of the molecular frameworks within these organic sensitizers is expected to refine key photophysical properties, including the absorption spectrum, photothermal conversion efficiency, and photostability. This refinement is poised to markedly augment the efficacy of organic–MXene heterojunctions in the realm of wound healing applications.

### Anti-inflammatory properties

The inflammatory response, a critical yet complex element in wound healing, serves as both a facilitator and a potential impediment [98,99]. A precisely regulated inflammatory phase is essential for the effective debriding of devitalized tissue and the neutralization of microbial threats, which are foundational to the wound healing sequence. However, excessive inflammation can derail the healing process by disrupting collagen synthesis, impeding angiogenesis, and disturbing the formation of granulation tissue. As our comprehension of inflammatory processes has matured, the focus has shifted toward the meticulous modulation of ROS levels and macrophage polarization within the wound environment to mitigate the risks associated with an overactive inflammatory response [94]. In this regard, MXenes have emerged as a subject of important research interest due to their unique characteristics. They have demonstrated the capacity to efficiently neutralize excessive ROS, thereby attenuating oxidative stress-induced cellular damage, and also possess the ability to influence macrophage polarization, which is pivotal in the resolution of inflammation [100].

#### Scavenging excessive ROS

The intracellular levels of ROS are stringently regulated, with their physiological concentrations playing a vital role in the clearance of pathogens and the removal of necrotic tissue at wound sites [101]. However, scenarios such as severe infection or hyperglycemia may provoke inflammatory cell infiltration, culminating in ROS overproduction and the onset of oxidative stress. This can result in substantial damage to cellular macromolecules, including DNA, proteins, and lipids [102]. If left unabated, the inflammatory response to tissue injury may progress into a chronic inflammatory cycle [103]. MXenes display dual functionality; they efficiently scavenge excess ROS to avert oxidative harm and facilitate the conversion of superoxide and H_2_O_2_ into O_2_, thereby alleviating hypoxia at the wound site. The exceptional efficacy of MXenes is attributed primarily to their unique mechanisms of electron and hydrogen atom transfer, coupled with their ability to emulate the functions of various endogenous antioxidant enzymes.

**Fig. 3.**
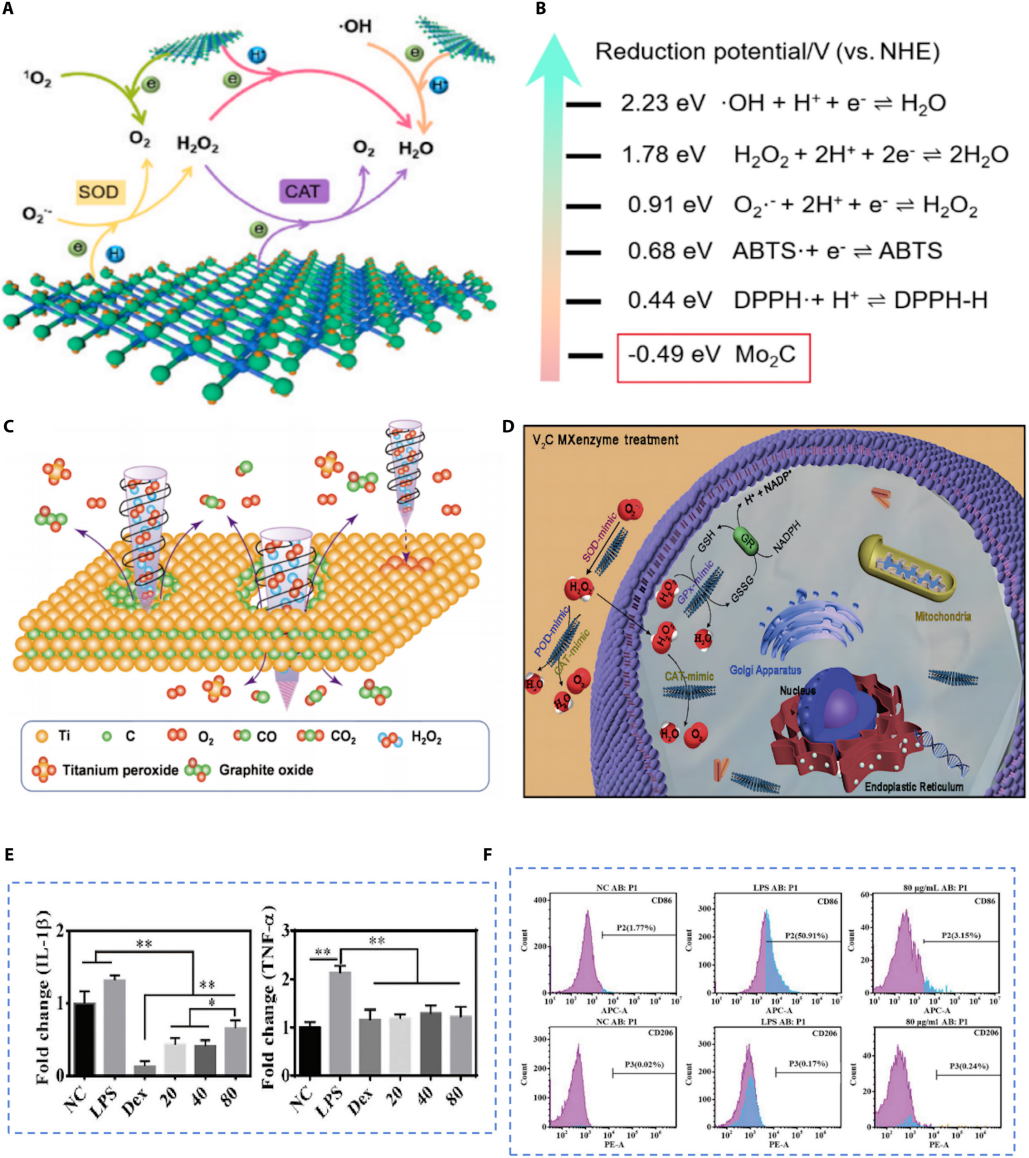
Anti-inflammatory mechanisms of MXenes. (A) Schematic illustration of the hypothesized antioxidant mechanism for Mo_2_C MXene. (B) Redox potential of Mo_2_C MXene and different free radicals. Reprinted from [100] with permission. Copyright 2024 Elsevier Ltd. (C) Illustration of the reaction mechanism of Ti_3_C_2_ MXene and H_2_O_2_. Reprinted from [104] with permission. Copyright 2022 Springer Nature. (D) Schematic representation of the ROS-scavenging capabilities of V_2_C MXene with multifaceted enzyme-mimicking attributes. Reprinted from [107] with permission. Copyright 2021 Springer Nature. (E). Comparative analysis of IL-1β and TNF-α gene expression in RAW264.7 cells across experimental groups. The groups denoted as 20, 40, and 80 correspond to the concentrations of Ti_3_C_2_ MXene at 20, 40, and 80 μg ml^−1^, respectively (*n* = 3, *P < 0.05, **P < 0.01). (F). Quantitative analysis of macrophage subtypes after 48 h of incubation with Ti_3_C_2_ MXene via flow cytometry. The proportion of M_1_ macrophages is displayed in the upper panel, with the M_2_ macrophages presented in the lower panel. Reprinted from [113] with permission. Copyright 2023 Wiley-VCH.

The interaction of MXenes with ROS initiates the active transfer of electrons or hydrogen atoms from their surface to ROS. During this process, MXenes undergo progressive oxidation, while ROS are efficiently reduced simultaneously, neutralizing their oxidative potential (Fig. 3A). A seminal study has reported that the standard electrode potential of Mo_2_C MXene is −0.491 eV, a value that underscores its robust reducing capacity in comparison to that of ROS, thereby endowing it with the capability to scavenge ROS efficiently (Fig. 3B) [100]. Liu et al. [104] delved into the kinetic details of the interaction between Ti_3_C_2_ MXene and H_2_O_2_. By utilizing a suite of characterization techniques to analyze the reaction products, they demystified the specific mechanisms involved (Fig. 3C). This investigation sheds light on the intricate dynamics between Ti_3_C_2_ MXene and H_2_O_2_, enhancing our comprehension of the fundamental processes at work.

The human body’s endogenous antioxidant defense system, comprising enzymes like SOD, catalase (CAT), and glutathione peroxidase (GPX), is integral to the neutralization of ROS and the alleviation of oxidative stress [105]. Despite its importance, this system can falter in effectively managing abnormal surges in ROS levels [106]. To address this challenge, Feng et al. [107] introduced the innovative “MXenzymes” concept, successfully creating the V_2_C MXenzyme. This novel MXenzyme mimics the functions of multiple endogenous antioxidant enzymes, adeptly catalyzing the transformation of ROS into harmless substances such as O_2_ within intricate biological systems (Fig. 3D). An ample supply of O_2_ fosters an oxygen-rich environment conducive to normal cellular physiological functions, thereby robustly supporting new tissue growth. The introduction of MXenzymes presents a revolutionary approach to regulate the metabolic imbalances of ROS in the wound microenvironment.

#### M_1_-to-M_2_ macrophage polarization promotion

The inflammatory environment at the wound site is highly dynamic, characterized by the ability of macrophages to polarize into the functionally distinct M_1_ and M_2_ phenotypes in response to various signals present within the wound microenvironment [108,109]. M_1_ macrophages are instrumental in pathogen elimination and the instigation of inflammatory responses; however, an unchecked release of their abundant pro-inflammatory cytokines may result in an overactive inflammatory state. Conversely, M_2_ macrophages are essential in the resolution phase of inflammation, where they foster wound healing by releasing cytokines and growth factors that enhance the restoration and regeneration of injured tissues [110–112].

In groundbreaking research, Li et al. [113] introduced an innovative strategy to modulate macrophage polarization by coculturing Ti_3_C_2_ MXene with M_1_ macrophages. The Ti_3_C_2_ MXene demonstrated a capacity to curb the overexpression of pro-inflammatory cytokines, such as interleukin-1β (IL-1β) and tumor necrosis factor-α (TNF-α), which are typically induced by LPS. Notably, its effect was found to be on par with that of dexamethasone (DEX), a widely utilized anti-inflammatory medication in clinical settings. Further analysis using flow cytometry revealed a substantial decrease in the expression levels of the M_1_ macrophage-specific surface marker CD86 (Fig. 3E and F). This study offers evidence that MXenes can modulate macrophage polarization, thereby exerting anti-inflammatory effects. Despite these promising findings, the precise mechanisms through which MXenes influence macrophage polarization are not yet fully understood, which may hinder a comprehensive grasp of their anti-inflammatory properties. Further research is essential to elucidate the intricate interactions between MXenes and macrophages, potentially laying the groundwork for novel and transformative therapeutic approaches.

### Angiogenesis

The ingrowth of new blood vessels is crucial for providing the necessary oxygen and nutrients required for cell proliferation and migration involved in tissue repair, for removing metabolic waste, and for facilitating the delivery of immune cells to combat potential infections. Furthermore, angiogenesis supports the growth and regeneration of new tissue, contributing to the restoration of skin structure and function [114]. In the realm of wound healing research, MXenes have garnered considerable attention, with a growing focus on understanding their role in regulating angiogenesis. To date, MXenes have been reported to modulate angiogenesis through various mechanisms, including ES and low photothermal induction.

#### Electrical stimulation

ES serves as an efficacious physical intervention that fosters cellular activities such as proliferation, migration, and differentiation. This is achieved by modulating intracellular signaling pathways and reconfiguring the intracellular microenvironment [115]. The application of vascular endothelial growth factor (VEGF) neutralizing antibodies can effectively promote angiogenesis, underlining the pivotal role of VEGF in angiogenesis [116]. The angiogenic responses elicited by ES are predominantly mediated by VEGF [117]. Additionally, ES is capable of directly inducing pre-angiogenic responses in vascular endothelial cells via VEGF receptor signaling. This suggests that endogenous electric fields may initiate essential angiogenic processes in vivo by activating VEGF receptor pathways.

The stratified architecture of MXenes supports the rapid transport of electrons within their layers, with the minimal interactions between layers creating a conductive pathway for electrons [118]. This feature renders MXenes an efficacious platform for ES-induced angiogenesis. These materials are endowed with a high specific surface area, which is conducive to functionalization with diverse groups, enhancing direct interactions with cells. The synergy of ES with MXenes leverages the benefits of both, allowing cells to receive ES signals more precisely and to react in a manner that modulates and bolsters cellular functions [119,120]. An avant-garde conductive hydrogel has been crafted by amalgamating regenerated bacterial cellulose (rBC) with Ti_3_C_2_ MXene (Fig. S3A) [118]. The integration of Ti_3_C_2_ MXene markedly augments the conductivity of the hydrogel, endowing it with the ability to serve as an electroactive matrix for electrical signal transmission within a wound bed and actively accelerating the in vivo wound healing process, as evidenced by the reduction in the wound area, and the enhancement in collagen synthesis, vascularization, granulation tissue formation, and re-epithelialization as well as gene expression of growth factors including VEGF, epidermal growth factor (EGF), and transforming growth factor-β (TGF-β) (Fig. S3B and C).

Nonetheless, it is imperative to acknowledge that the voltage level of the applied ES markedly affects the proliferation of NIH3T3 cells, which in turn influences angiogenesis. Ren et al. [121] conducted an in-depth study to observe changes in NIH3T3 cell behavior by varying the intensity of ES in the presence of Ti_3_C_2_ MXene. Within the range of 0 to 200 mV, cell proliferation is positively correlated with the intensity of ES. However, when the voltage reaches 400 mV, the cell’s proliferative capacity begins to be inhibited (Fig. S3D). This underscores that moderate ES can augment the proliferative vigor of NIH3T3 cells, whereas an overly intense ES may impede cell survival. These insights are instrumental in strategizing the application of MXene conductivity to foster angiogenesis.

#### Low-temperature PTT

Low-temperature PTT, applied within the 40 to 43 °C range, has been demonstrated to stimulate the proliferation of vascular endothelial cells, effectively augmenting vascular density within granulation tissue [122,123]. In a study conducted by Qu et al. [124], human umbilical vein endothelial cells (HUVECs) were treated with various concentrations of Ti_3_C_2_ MXene and subsequently exposed to a 10-min laser irradiation regimen. The temperature of cells treated with MXene nanosheets of various concentrations all increased to below 45 °C, effectively inducing a mild PTT effect and significantly up-regulating the expression of VEGF and CD31 (Fig. S3E and F). More importantly, the cell viability of cells treated with Ti_3_C_2_ MXene at different concentrations after NIR laser irradiation was above 95%, indicating that the moderate-temperature photothermal MXene has no effect on the proliferation of HUVECs. In ex vivo wound healing assays, cells subjected to MXene treatment and NIR laser irradiation displayed accelerated wound closure rates relative to control groups. This research underscores the salutary influence of an optimized photothermal effect on angiogenesis, potentially due to PTT-induced enhancements in blood circulation and amelioration of the hypoxic microenvironment.

In summary, in the initial stage of wound healing, MXenes effectively inhibit bacteria and reduce the risk of infection through PDT, PTT, and nanoknife technology, laying a solid foundation for smooth wound healing. As the wound enters the inflammatory phase, they can scavenge excess ROS while inducing macrophage polarization toward the M_2_ phenotype, thereby effectively alleviating inflammatory responses and promoting tissue regeneration. In the later stage of wound healing, MXenes utilize their unique photothermal effect and excellent conductive properties to importantly accelerate the formation of new blood vessels, ensuring adequate blood supply and nutritional support to the wound area, which provides powerful assistance for comprehensive tissue repair. These mechanisms work synergistically to efficiently drive the wound healing process forward.

## Current State of Research on MXene-Based Multifunctional Bioactive Composite Materials in Wound Healing

MXenes demonstrate marked promise in wound healing due to their unique physicochemical and biological properties. However, to maximize their clinical potential, several challenges must be addressed [7]. One major challenge is the lamellar structure of MXenes, which struggles to adapt to the complex and irregular shapes of wounds. Moreover, this structure is prone to oxidation, self-aggregation, and accumulation during therapeutic use, potentially diminishing their therapeutic impact [125]. Fortunately, combining MXenes with other bioactive materials in composite forms has emerged as a promising strategy to tackle these issues [126,127]. In the following section, we will conduct a thorough and systematic review of the current literature on these composites, focusing on their applications in wound healing.

### MXene–Hydrogel Composite Materials

The 3D porous network structure of hydrogels endows them with exceptional water absorption and retention capabilities, making them highly favored in fields such as drug delivery, tissue engineering, and wound dressings [128–130]. By integrating with MXenes, the performance of these hydrogels is markedly enhanced. The incorporation of MXenes not only enhances the mechanical strength of hydrogels but also improves their transport efficiency. Conversely, the porous structure of the hydrogel provides an ideal dispersion platform for MXenes, effectively preventing aggregation due to mutual attraction and ensuring their uniform dispersion. Moreover, encapsulating MXenes within hydrogels leverages the shape adaptability of hydrogels, enhancing the adaptability of MXenes and allowing them to conform to wounds of various shapes and irregularities.

**Fig. 4.**
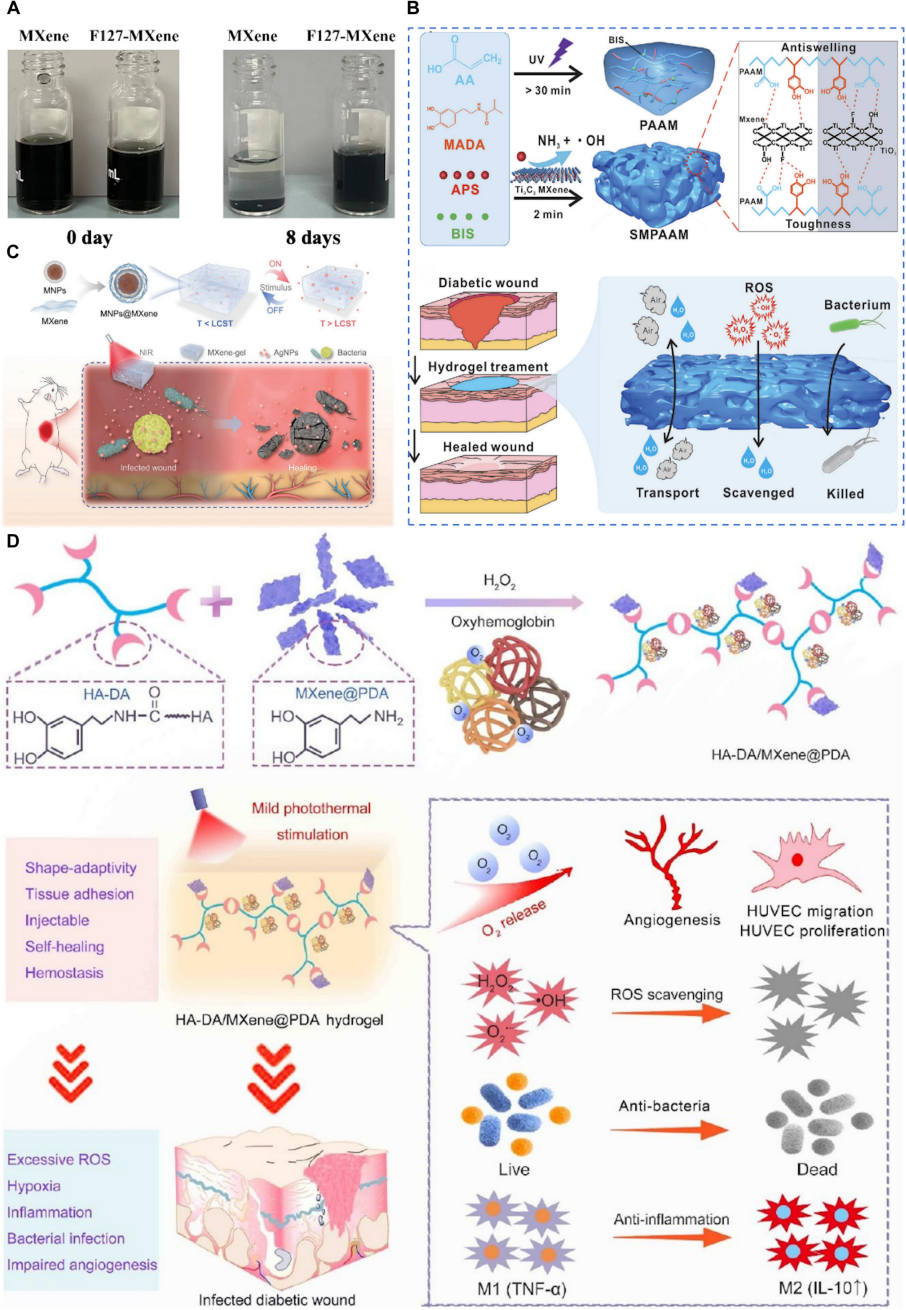
MXene*–*hydrogel composite materials. (A) State of the F127–MXene hydrogel and Ti_3_C_2_ MXene solution on days 0 and 8. Reprinted from [131] with permission. Copyright 2024 American Chemical Society. (B) Design strategy for the sponge-like macro-porous hydrogel system. Reprinted from [133] with permission. Copyright 2022 Wiley-VCH. (C) Schematic illustration of the fabrication and utilization of a stimuli-responsive MXene*–*hydrogel system. Reprinted from [134] with permission. Copyright 2021 Wiley-VCH. (D) Schematic illustrations of injectable HA-DA/MXene@PDA hydrogel preparation. Reprinted from [135] with permission. Copyright 2022 American Chemical Society.

The integration of hydrogels enhances the application of MXenes in wound healing. Initially, hydrogels facilitate the even dispersion of MXenes across the wound surface, enhancing their stability. Jiang et al. [131] successfully embedded Ti_3_C_2_ MXene into an F127 hydrogel matrix, creating an F127–MXene composite. Under open oxygen conditions, pure Ti_3_C_2_ MXene solutions undergo severe oxidation and degradation within 8 days, leading to significant aggregation and precipitation. However, when Ti_3_C_2_ MXene is embedded within the F127 hydrogel system, it maintains a uniform dispersion and exhibits remarkable stability (Fig. 4A). Furthermore, when MXenes are encapsulated within hydrogels, they can conform to wounds of various shapes and sizes, including irregular ones, and enhance their retention time on the wound bed. For instance, hydrogels formed in situ through a photo-crosslinking mechanism, emulating the adhesion strategy of mussels, increase the residence time on wound tissues, thereby augmenting the dispersion and retention time of MXenes at irregular wound sites [132].

The flexibility and hydrophilicity of MXenes also provide an effective way to regulate the wound microenvironment. Wei et al. [133] created an innovative sponge-like hydrogel named SMPAAM by mixing Ti_3_C_2_ MXene with acrylic acid (AA), dopamine methacrylate (MADA), ammonium persulfate (APS), and *N*,*N*′-methylenebisacrylamide (BIS) (Fig. 4B). This hydrogel has a robust macroporous structure with pore sizes of 200 to 300 μm, offering optimal mass and nutrient permeability. The incorporation of Ti_3_C_2_ MXene significantly enhances the water and blood transport capabilities of the SMPAAM hydrogel, which is about 20 times faster than that of nonporous hydrogels. Furthermore, the strong interaction between Ti_3_C_2_ MXene and the polymer chains of the hydrogel endows SMPAAM with excellent anti-swelling properties in various media. Given the good photothermal conversion efficiency of MXenes, the hydrogel can be endowed with a unique light-stimulated drug release function. Yang et al. [134] prepared an injectable hydrogel that responds to NIR light, allowing for flexible regulation of therapeutic drug release by applying or removing external NIR irradiation. This method can achieve remote, noninvasive manipulation of cellular behavior (Fig. 4C).

Integrating MXenes with hydrogels to combine their advantageous properties represents a promising frontier in research. Existing studies have demonstrated the immense potential of composite materials formed by MXenes and hydrogels in accelerating wound healing. Li et al. [135] created a thermo-responsive oxygen delivery system by integrating polydopamine (PDA)-coated Ti_3_C_2_ MXene and oxyhemoglobin (HbO_2_) with hyaluronic acid-grafted dopamine (HA-DA) hydrogel, which facilitates synergistic healing of diabetic wounds (Fig. 4D). The combination of HA-DA hydrogel with MXenes shows a stronger effect in accelerating wound healing than monotherapy. This is attributed to the PDA coating enhancing the antioxidant and antibacterial capabilities of MXene, as well as promoting the crosslinking of MXene nanosheets into a hydrogel network. Moreover, the mild PTT effect generated by MXenes under laser irradiation not only directly stimulates cell proliferation and migration but also indirectly down-regulates hypoxia-inducible factor-1α (HIF-1α) and VEGF by activating oxygen carriers.

The forthcoming research on MXene-hydrogel systems is on the cusp of ushering in a new era distinguished by multifunctional integration and intelligent design. By meticulously replicating the intricate milieu of natural tissues and crafting material compositions with precision, the scientific community is dedicated to the innovation of smart biomaterials. These materials are endowed with the capacity to sense and respond to external stimuli, embodying the potential for environmental detection, self-regulation, and adaptive repair mechanisms. Such intelligent materials are envisioned to provide more tailored and precise therapeutic interventions for wound healing.

### MXene–MN composite materials

MNs have emerged as a compelling drug delivery platform due to their ease of administration, versatile drug-loading capacity, and tunable release profiles [136,137]. These systems typically comprise an array of slender, micrometer-scale projections that can painlessly penetrate the surface of skin, bypassing the protective barriers such as blood clots, scars, and exudates at wound sites, thus enabling targeted medication delivery to the affected region [138,139]. However, the efficacy of MN-based drug delivery systems is seriously influenced by factors like dissolution kinetics and mechanical integrity [140,141]. To surmount these challenges, researchers have spearheaded an innovative approach that integrates MNs with MXenes, presenting a promising solution to enhance drug delivery efficacy.

**Fig. 5.**
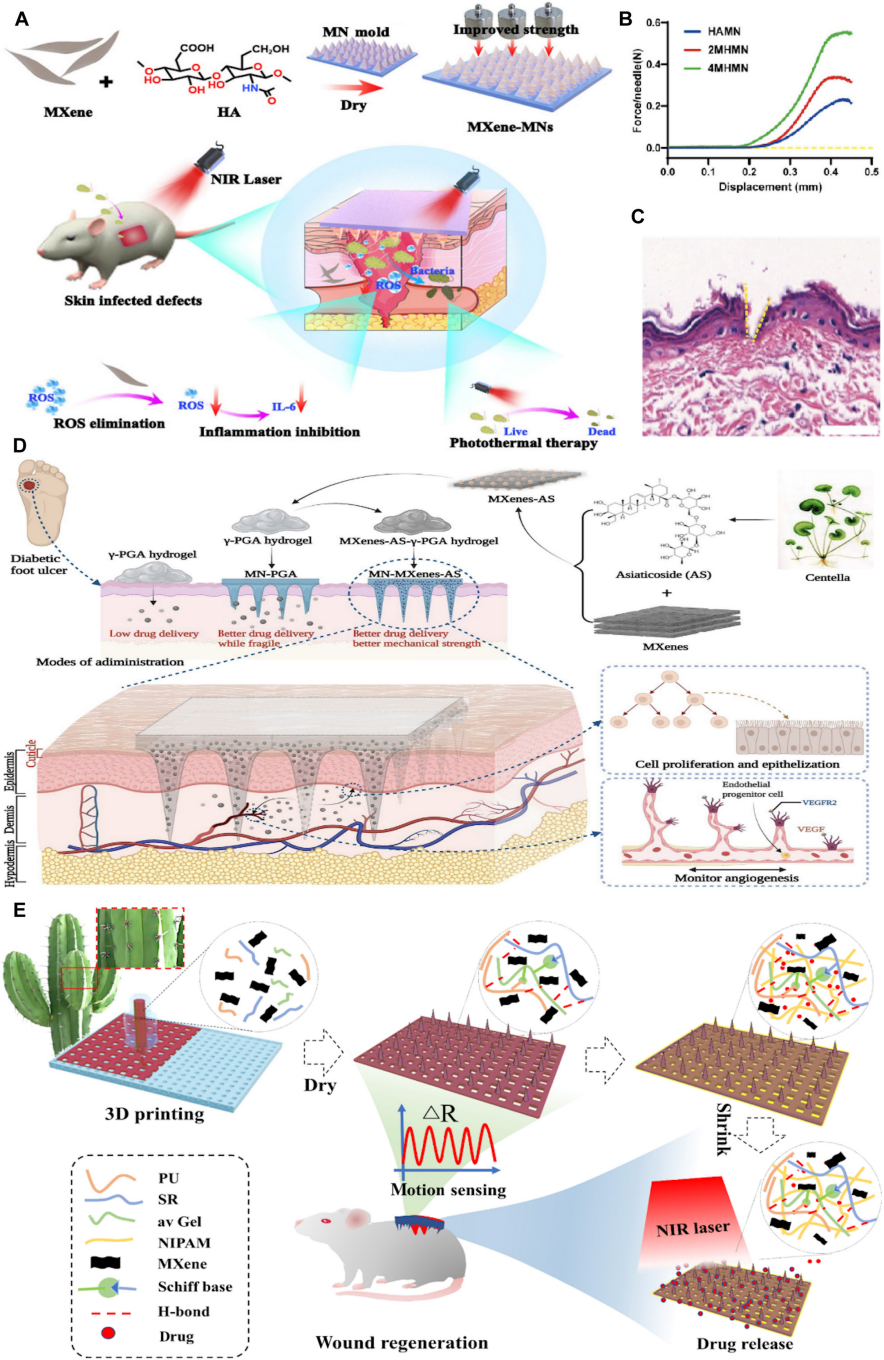
MXene*–*MN composite materials. (A) Schematic representation of Ti_3_C_2_ MXene-reinforced MN patches: Harnessing photothermal antibacterial effects and ROS neutralization for enhanced infected wound healing. (B) Comparative analysis of the mechanical properties of the HAMN, 2MHMN, and 4MHMN formulations. HAMN: HA MN; 2MHMN: 200 μg ml^−1^ Ti_3_C_2_ MXene + HA MN; 4MHMN: 400 μg ml^−1^ Ti_3_C_2_ MXene + HA MN. (C) Hematoxylin and eosin (H&E) staining of skin sections after MN treatment. Scale bar: 50 μm. Reprinted from [144] with permission. Copyright 2023 Royal Society of Chemistry. (D) Schematic illustration of MXenes-based MN patch for accelerating diabetic wound healing. Reprinted from [147] with permission. Copyright 2022 Springer Nature. (E) Schematic illustration of 3D-printed bionic NIR light-responsive MXene and spidroin-based MN scaffolds for skin wound healing. Reprinted from [149] with permission. Copyright 2022 American Chemical Society.

The superior mechanical properties of MXenes substantially reinforce the tensile strength of MN composites, enhancing their durability and reliability for practical use. Importantly, this integration retains the inherent biological activities of MXenes [142,143]. Zhong et al. [144] reported a multifunctional MXene–MN composite material, composed of hyaluronic acid (HA) and Ti_3_C_2_ MXene (Fig. 5A). In addition to the inherent mechanical properties of Ti_3_C_2_ MXene, the hydrophilic chemical groups (-OH and -F) on the surface of Ti_3_C_2_ MXene can be physically crosslinked with HA molecules through interactions such as hydrogen bonding, thereby enhancing the mechanical strength of the composite material. Compared to blank MNs, MN doped with Ti_3_C_2_ MXene exhibits the maximum compressive load capacity (up to 0.55 N per needle) at the same displacement distance, which is much greater than the force required to penetrate the skin (Fig. 5B and C). Therefore, MXene-enhanced MNs possess strong permeability, capable of rapidly piercing the stratum corneum of the skin and releasing Ti_3_C_2_ MXene nanosheets in the dermis. Under NIR irradiation, MXene–MNs exhibit an enticing photothermal bactericidal effect, markedly curbing the growth and proliferation of drug-resistant bacteria in the wound environment. Moreover, MXene-doped MNs can further reduce the expression of pro-inflammatory factors by scavenging ROS, accelerating neovascularization and soft tissue regeneration, thereby promoting the healing of infected wounds.

The integration of additional bioactive agents into the MXene–MN composite material amplifies its wound healing efficacy. Asiaticoside (AS), an organic compound, is considered the main therapeutic agent for promoting cell proliferation and monitoring angiogenesis during wound healing in patients with diabetes [145,146]. Wang and his team [147] devised an innovative therapeutic intervention for diabetic wound care by co-encapsulating Ti_3_C_2_ MXene and AS within poly-γ-glutamic acid (γ-PGA) hydrogel MNs, designated MN-MXenes-AS (Fig. 5D). Ti_3_C_2_ MXene bolsters the mechanical integrity of the MN composite material and concurrently functions as a carrier for the sustained delivery of AS, effectively prolonging its release profile. Comparative assessments demonstrated that the MN-MXenes-AS group surpassed the MN-MXenes groups in augmenting cellular proliferation and angiogenesis. In another study by Wang and colleagues [148], they encapsulated glucose oxidase (GOx)-loaded Ti_3_C_2_ MXene nanosheets within MN patches composed of γ-PGA (MN-PGA-MXene-GOx). GOx, which lowers local glucose and pH by oxidizing glucose, is delivered deep into tissues via the MN patch, while MXene ensures its prolonged release. When the MN patch is exposed to external NIR light, MXene can induce mild hyperthermia, enhancing the catalytic activity of GOx and promoting cell proliferation, migration, angiogenesis, and tissue remodeling. Both γ-PGA and MXene possess antioxidant properties that can alleviate ROS in the diabetic environment and reduce the production of H_2_O_2_ during glucose oxidation. These studies offer a pioneering strategy and direction for diabetic wound therapy, highlighting the vast potential of interdisciplinary convergence between biomaterials and pharmacotherapy.

Most traditional MN patches struggle to achieve precise control over drug release. This is primarily because they rely on the degradation rate of the material to trigger drug release, a method that finds it challenging to exert exact control over time and space. In light of this, Shao et al. [149] have utilized 3D printing technology to prepare a MN scaffold with photothermal responsive drug release and self-healing properties by blending polyurethane (PU), aloe vera gel (av Gel), MXene, and silk protein (SR), for the promotion of wound healing (Fig. 5E). The superior electrical and photothermal properties of MXene allow the MN scaffold to conduct sensitive wound motion monitoring and controlled drug release under NIR radiation. Additionally, inspired by the growth of cactus spines, the inverse opal (IO) photonic crystal (PC) structure of the MNs and the ample space between the scaffold struts endow the MN scaffold with high drug-loading capacity. The temperature-responsive *N*-isopropylacrylamide (NIPAM) pre-gel, containing the antibiotic mupirocin and human EGF (hEGF), permeates into the MNs’ IO PC structures and the interstitial spaces of the scaffold. The drug-loaded MN scaffold can slowly release its contents without compromising its own structure under NIR irradiation, due to the photothermal conversion of MXene and the volume contraction of NIPAM with increasing temperature. These unique characteristics indicate that the multifunctional MN scaffold based on MXene shows great potential in promoting wound healing and will be widely used in wound management.

Although research on MXene–MN composite nanomaterials for wound healing is still in its infancy, their considerable promise and potential highlights the necessity for continued exploration. To refine the delivery strategy of MXenes, MNs can be engineered into diverse structural configurations to meet a range of therapeutic demands [150–152]. For example, MNs can be crafted with a multi-zone structure, where each zone is tailored with unique drug-loading and release profiles, thus enhancing their adaptability to the intricate dynamics of the wound environment [153].

### MXene–hemostatic sponge composite materials

In the field of wound healing, rapid hemostasis is a critical metric for dressing performance [154–156]. Hemostatic sponges can rapidly absorb blood and form a stable gelatinous substance within a short timeframe, thus achieving efficient hemostasis. This unparalleled hemostatic capability, combined with good biocompatibility and nonirritant properties, makes it the material of choice for hemostasis in wound management [157,158].

Chitosan sponges, known for their soft texture, excellent permeability, and rich microporous structure, effectively absorb wound exudates while providing an optimal environment conducive to cell growth [159–161]. MXenes, with their exceptional photothermal antimicrobial properties, coupled with high hydrophilicity and specific surface area, expedite the absorption of plasma moisture and concentrate coagulation factors. These attributes render MXenes as promising enhancers for boosting the antimicrobial and hemostatic capabilities of chitosan sponges. In a groundbreaking study, researchers developed a multifunctional sponge that offers hemostasis, sustained antimicrobial action, and promotion of angiogenesis by integrating surface-modified Ti_3_C_2_ MXene, Cu^2+^, and chitosan (CMN-Cu) (Fig. S4A) [162]. Upon NIR irradiation, the synergistic effect of the photothermal properties of the Ti_3_C_2_ MXene and the release of Cu^2+^ markedly intensified the antimicrobial efficacy of the chitosan sponge (Fig. S4B). A mouse liver injury model demonstrated that the groups containing Ti_3_C_2_ MXene, particularly CMN-Cu and CMN, outperformed the plain chitosan sponge in terms of hemostatic performance (Fig. S4C). The creation of this innovative multifunctional sponge has notably advanced our understanding of state-of-the-art hemostatic materials. With its sophisticated design and multifaceted functionalities, this sponge is set to redefine the standards for hemorrhage control and wound care.

The hydrophilicity, specific surface area, and functional group composition of MXenes are pivotal attributes that can profoundly affect their performance as enhancers. Li et al. [163] conducted an in-depth investigation into how different morphologies of Ti_3_C_2_ MXene—presented as accordion-like (MX), intercalated (iMX), single-layer (sMX), and single-layer decorated with gold nanoparticles (Au@sMX)—impact the efficacy of chitosan hemostatic sponges (CHs) (Fig. S4D). The findings of this study underscored that the morphology of Ti_3_C_2_ MXene exerts a substantial influence on the mechanical, antimicrobial, and hemostatic properties of CHs. Notably, the CH/Au@sMX composite demonstrated synergistic aggregation of all the aforementioned superior characteristics, facilitating rapid hemostasis, resisting infection, and promoting skin regeneration, thereby positively contributing to the healing process of infected skin wounds. Further exploration of the mechanisms underlying the action of MXenes with varying morphologies, coupled with the optimization of their conditions, paves the way for the development of hemostatic materials that are not only better performing but also safer and more reliable, addressing the pressing demands of clinical practice.

### MXene–nanofibrous membrane composite materials

Nanofiber membranes (NFMs) are widely recognized for their large specific surface area, high porosity, and spatial interconnectivity, which render them highly advantageous for nutrient transport, cell communication, and eliciting effective cell responses [164].

Despite these commendable properties, NFMs are not fully equipped to address the complex challenges presented by the wound healing microenvironments. A marked limitation is their capacity to effectively combat bacterial infections, modulate inflammation, and promote tissue regeneration [165,166]. MXenes possess unique physicochemical properties that complement the disadvantages of NFMs. Deng and colleagues [167] constructed a composite material with photodynamic and photothermal effects by integrating MXenes with optical properties into NFMs composed of polycaprolactone (PCL). Furthermore, the composite material was supplemented with the anti-inflammatory drug aspirin to regulate the levels of ROS. Experimental results indicate that the generation of ROS and the combination with aspirin can effectively kill bacteria and counteract inflammation, thereby promoting the wound healing process (Fig. S4E). Excitingly, leveraging the exceptional conductivity of MXenes, Zhao and colleagues [168] have successfully developed a composite NFM that actively responds to physiological electrical signals. This innovative material skillfully integrates the superior mechanical properties of PCL, the biocompatibility of gelatin, and the electroactive characteristics of Ti_3_C_2_ MXene (MPG). Under the influence of ES, this scaffold notably enhances the adhesion, proliferation, and migration capabilities of NIH3T3 cells. Furthermore, in vivo assessments using a full-thickness wound defect model have demonstrated the MPG-6 membrane’s remarkable efficacy: it not only accelerates wound closure but also promotes granulation tissue formation, increases collagen deposition, and effectively advances wound vascularization (Fig. S4F).

In summary, the spatial interconnectivity of NFMs combined with the unique properties of MXenes creates a highly favorable microenvironment for cell communication and the elicitation of effective cellular responses, ultimately accelerating tissue regeneration and wound healing. The integration of MXenes into NFMs represents an important advancement in wound healing technology, providing a multifaceted approach to address the complex challenges associated with the wound microenvironment.

## Conclusion, Challenges, and Future Perspectives

This article provides a comprehensive review of the therapeutic mechanisms of MXenes in wound healing, encompassing their physical, photothermal, and photodynamic effects on bacteria, as well as their roles in reducing inflammation, promoting angiogenesis, and supporting tissue regeneration. The review also discusses the recent progress in MXene-based composites for accelerating wound healing. The strategic integration of MXenes with nanomaterials, including hydrogels, MNs, and hemostatic sponges, not only overcomes their intrinsic limitations but also enhances the therapeutic efficacy of the composites. This advancement expands the horizons for MXenes in fostering wound care and healing therapies.

While MXene-based bioactive materials offer remarkable potential for wound healing, several challenges must be addressed. Initially, the application of MXenes in this domain has been predominantly centered on titanium-based carbides. However, the MXene family is expansive and includes niobium-, vanadium-, and molybdenum-based carbides or nitrides, each with unique physicochemical properties that could pioneer innovative therapeutic approaches for wound management. There is an urgent need for researchers to broaden the spectrum of MXenes and explore their potential for wound healing through interdisciplinary research endeavors. Second, the use of the PTT effect of MXenes for bacterial inactivation raises concerns, as the generation of localized high temperatures may endanger surrounding healthy cells and tissues. Employing a low-temperature PTT strategy can circumvent this thermal risk and also promote angiogenesis and collagen deposition. Concurrently, optimizing the nanostructure and surface features of MXenes to enhance their ability to selectively target bacteria can mitigate unintended thermal impacts on nontarget cells or tissues. Moreover, the PTT and PDT effects of MXenes might drive macrophage polarization toward the pro-inflammatory M_1_ phenotype, potentially worsening the inflammatory response. An effective countermeasure to address this issue could involve the integration of MXenes with antioxidants, which could strategically employ their antioxidant properties to modulate the inflammation heightened by the PTT and PDT effects of MXenes. Ultimately, enhancing the encapsulation efficiency and precision of drug delivery for MXene-based composite materials represents a critical challenge. Tackling this requires refining the design strategies of current composite materials, venturing into cutting-edge bio-nanomaterials, and innovating in the development of stimuli-responsive drug release mechanisms. These strategies can enable the sustained and precise release of MXenes at the wound site, thereby markedly amplifying the efficacy of the wound healing process.

Research on MXenes is projected to concentrate on the following pivotal directions: (a) Developing cost-effective and efficient synthesis techniques for MXenes to enable their extensive application in the biomedical field. (b) Long-term evaluation of the in vivo biocompatibility and biodegradability of MXenes could provide a solid scientific foundation for their clinical application. (c) The stability of MXenes is a prerequisite for their effective biological function, making the development of new types of MXenes with enhanced environmental stability an essential pursuit. (d) Enhancing the bacterial selectivity of MXenes is a pivotal research focus, which entails surface modification and functionalization of MXenes to ensure both excellent biocompatibility and potent antimicrobial activity. (e) In-depth investigations of the specific mechanisms by which MXenes exert antimicrobial and anti-inflammatory effects and promote angiogenesis, as well as studies of the synergistic effects among these mechanisms, are needed to further enhance the therapeutic efficacy of MXenes. (f) The integration of MXenes with other bioactive materials aims to develop intelligent and personalized wound treatment strategies that cater to the unique needs of diverse patient populations.The in-depth exploration of these research directions aims to fully explore the application potential of MXenes in the field of wound healing, with the expectation of offering more efficient and safe treatment options for the field.

## Data Availability

Data sharing is not applicable to this article as no datasets were generated or analyzed during the current study.
