# Supplementary material for: MXene-Derived Multifunctional Biomaterials: New Opportunities for Wound Healing
Source: Biomater Res. 2025 Feb 11;29:0143. doi: 10.34133/bmr.0143 (PMC11811641; doi:10.34133/bmr.0143)
Supplement: Supplementary 1 — Figs. S1 to S4 Table S1 [file bmr.0143.f1.docx]

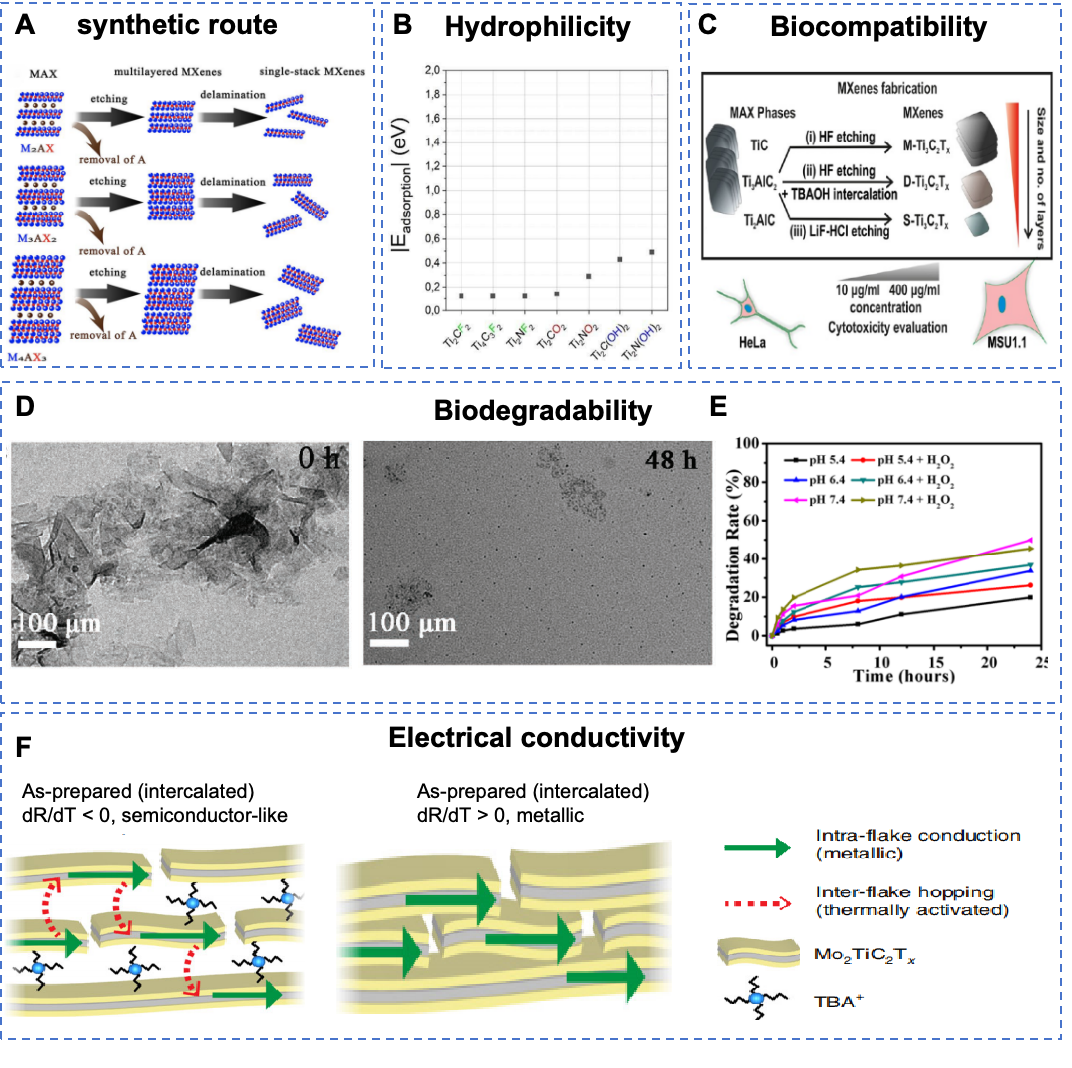


**Fig. S1.** Synthesis methods and properties of MXenes (A) Structure of MAX phases and the corresponding MXenes. Reprinted from Ref. [29] with permission. Copyright 2023 Springer Nature. (B) Absolute values of the adsorption energy of one single water molecule on different types of MXenes. Reprinted from Ref. [41] with permission. Copyright 2023 Springer Nature. (C) Schematic representation of the biocompatibility study of different sizes and concentrations of Ti_3_C_2_ MXene. Reprinted from Ref. [43] with permission. Copyright 2019 American Chemical Society. (D) TEM images of the degradation product of Mo_2_C-PVA incubated in PBS (pH 7.4) at 0h and 48h. (E) The degradation rate of Mo_2_C-PVA nanoflakes after incubating various pH values of PBS containing 500 ppm H_2_O_2_ or RPMI 1640 medium containing 500 ppm H_2_O_2_ and 10% FBS. Reprinted from Ref. [46] with permission. Copyright 2019 Wiley-VCH. (F) Schematic of intercalants’ influence on conduction through multi-flake MXenes. Reprinted from Ref. [48] with permission. Copyright 2019 Springer Nature.

**
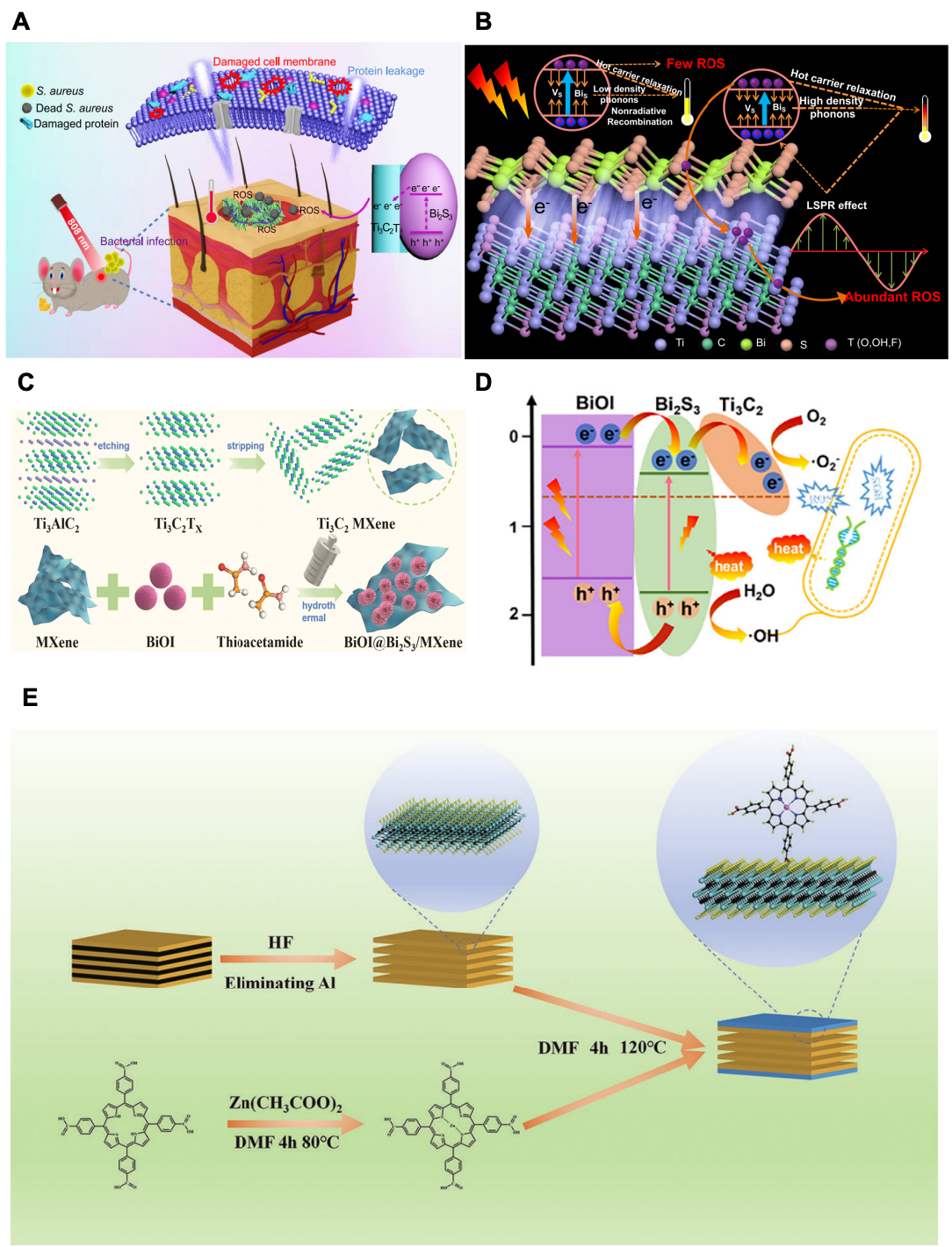
**

**Fig. S2**. Enhanced PTT and PDT effects of MXene heterojunctions. (A) The schematic illustration of Bi_2_S_3_/Ti_3_C_2_T_x_ schottky catalyst and antibacterial mechanism diagram of Bi_2_S_3_/Ti_3_C_2_T_x_ under 808 nm irradiation. (B) Schematic diagram for photodynamic and photothermal mechanism between Ti_3_C_2_T_x_ and Bi_2_S_3_. Reprinted from Ref. [94] with permission. Copyright 2021 Spring Nature. (C) Schematic illustration of the synthesis of BiOI@Bi_2_S_3_/Ti_3_C_2_. (D) Schematic illustrations of the PTT/PDT mechanism of BiOI@Bi_2_S_3_/Ti_3_C_2_. Reprinted from Ref. [95] with permission. Copyright 2023 Elsevier Inc. (E) Synthesis of ZnTCPP/Ti_3_C_2_T_X_. Reprinted from Ref. [85] with permission. Copyright 2022 Wiley-VCH.


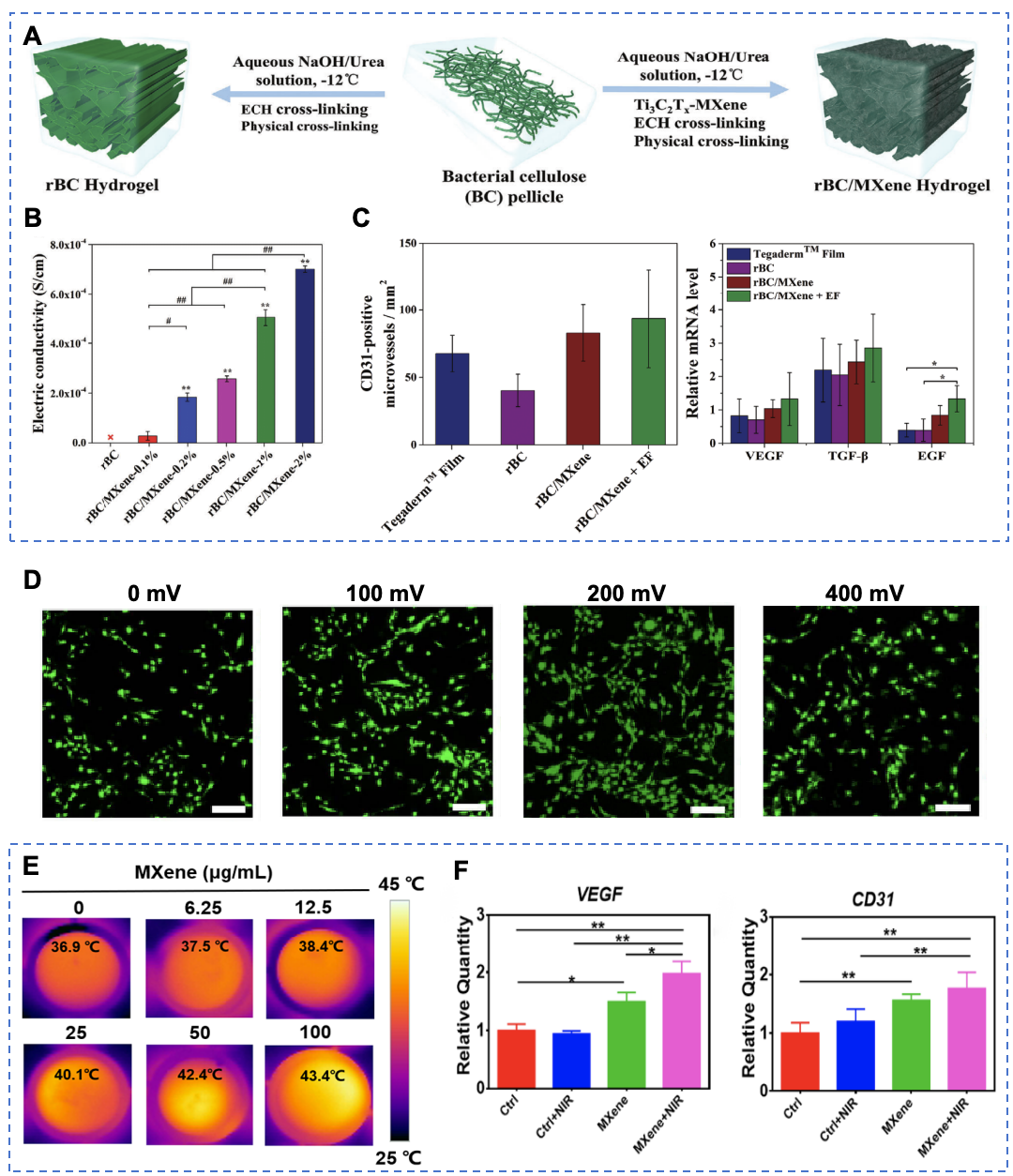


**Fig. S3.** The mechanism by which MXenes promote angiogenesis. (A) Schematic sketch showing the fabrication of rBC-based hydrogels. (B) The electric conductivity of rBC-based hydrogels. (C) Quantification of CD31-positive microvessels and analysis of gene expressions of TGF-𝛽, EGF, and VEGF growth factors on day 14 after different treatments. Reprinted from Ref. [118] with permission. Copyright 2020 Wiley-VCH. (D) The cell survival under different voltages. Scale bar: 200 μm. Reprinted from Ref. [121] with permission. Copyright 2024 Dove Medical Press Ltd. (E) Infrared thermographic analysis of HUVECs post-exposure to laser irradiation (808 nm, 0.75 W/cm²) with varying concentrations of Ti_3_C_2_ MXene nanosheets. (F) Relative gene expression levels of VEGF and CD31 in HUVECs following exposure to diverse treatments (n = 3, *p < 0.05, **p < 0.01). Reprinted from Ref. [124] with permission. Copyright 2023 American Chemical Society.


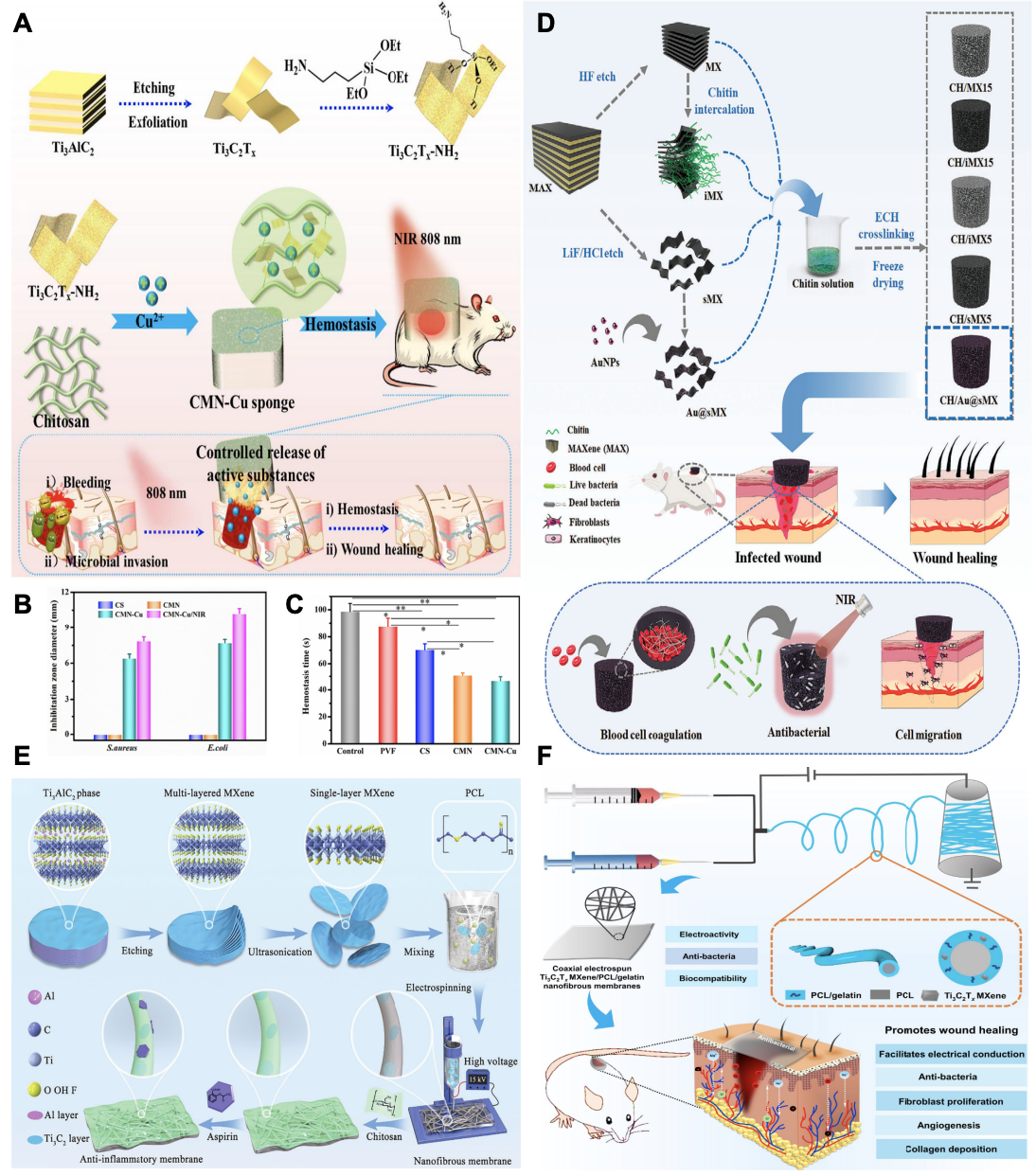


**Fig. S4.** MXene***–***hemostatic sponge composite materials. (A) Schematic representation of the fabrication and application process for the CMN-Cu sponge. (B) Inhibition zone diameter of the different sponges. (C) Hemostatic time of the different sponges in the rat liver injury model (n = 5, *p < 0.05, **p < 0.01). PVF: poly(vinyl formal); CMN: CS + Ti_3_C_2_ MXene; CMN-Cu: CS + Ti_3_C_2_ MXene + Cu^2+^. Reprinted from Ref. [162] with permission. Copyright 2023 Elsevier B.V. (D) Schematic overview of chitin/MXene composite sponges for the management of bacterium-infected wounds. Reprinted from Ref. [163] with permission. Copyright 2021 Wiley-VCH. (E) the preparation of the MXene-decorated nanofibrous membrane Reprinted from Ref. [167] with permission Copyright 2023 Wiley-VCH. (F) Schematic diagram of preparation, characteristics, and application of the electroactive and antibacterial Ti_3_C_2_T_x_ MXene/PCL/gelatin coaxial nanofibrous membranes. Reprinted from Ref. [168] with permission Copyright 2023 Spring Nature.

**Table S1**

Different synthesis methods for MXenes and their benefits and limitations.

| **Methods** | **Etching agents** | **Advantages** | **Disadvantages** | **Ref.** |
| --- | --- | --- | --- | --- |
| Fluoride solution etching | HF  LiF/NaF/ KF + HCl  HF/HCl | 1.High yield  2.Convenient operation | 1.Corrosive and polluting the environment  2.Uneven etching depth | [30, 31] |
| Alkali etching | NaOH  TMAOH | 1.Fluorine-free functional group  2.No risk of acid corrosion | 1.Etching conditions demanding  2.Low etching precision | [32, 33] |
| Molten salt etching | LiF/NaF/KF | 1.Even etching  2.Obtaining nitride MXenes | 1.Produce impurities  2.Poor crystallinity | [34] |
| Electrochemical etching | NH_4_Cl  + TMAOH | 1.High efficiency  2.Even etching | 1.Low yield  2.High equipment requirements  3.Difficult to operate | [35] |
| Chemical vapor  Deposition (CVD) | Methane and bimetal foil (Cu/Mo) | 1.High purity  2.Can be directly synthesized into MXenes | 1.Low yield  2.High equipment  requirements  3.Difficult to operate | [36, 37] |
